# *Hibiscus esculentus* and diabetes mellitus

**Published:** 2015-11-01

**Authors:** Fatemeh Dehghan Shahreza

**Affiliations:** ^1^Department of Clinical Science, School of Veterinary Medicine, Ferdowsi University of Mashhad, Mashhad, Iran

**Keywords:** *Hibiscus esculentus*, Diabetes mellitus, Hyperglycemia

**Implication for health policy/practice/research/medical education:**

Diabetes mellitus is a chronic metabolic disease, and is an important challenge of healthcare system in throughout the world. The most efforts are directed to control dyslipidemia and hyperglycemia in diabetes patients. Amongst various antidiabetic plants, recent investigations have shown favorable finding regarding Abelmoschus esculentus efficiency on improvement of blood glucose control and lipid profile abnormalities.

## Introduction

Diabetes mellitus is a chronic metabolic disease which involves nearly all organs of human body, and is an important challenge of healthcare system in throughout the world. It is a prominent risk factor for cardiovascular disorders such as hypertension and atherosclerosis, renal failure, neuropathies, eye and skin complications. Hyperglycemia induced over production of reactive species and pro-inflammatory processes are responsible to change normal cellular structure and function, which finally leads to endothelial cell dysfunction and cell death. Therefore, the most efforts are directed to reduce dyslipidemia and hyperglycemia as the diabetic complications (1). By administrating appropriate medication and correction of lifestyle, dyslipidemia and hyperglycemia are manageable. In addition it has been suggested, synthetic medications administration sometimes can complicate deficient cellular metabolic state. In this regard, administration of various plants and fruits are recommended due to containing natural bioactive components that are efficient to improve diabetic abnormalities. Antidiabetic plants can lower blood glucose through multiple mechanisms including; revival damaged Langerhans islet β-cells that lead to enhance insulin release, enhancement of glucose transporters and insulin receptor sensitivity, reduction of polysaccharides ingesting enzymes in intestinal tract, inhibition gluconeogenesis and *glycogenolysis (2) *.

Amongst various antidiabetic plants, recent investigations have shown favorable finding regarding *Abelmoschus esculentus* efficiency on improvement of blood glucose control and lipid profile.

*Abelmoschus* *esculentus,* okra,**of mallow family is a flowering plant, native to Southern Europe, Asia and Africa. It has been known as healthy plant; have wide range medicinal activity mediated by its active constituents. It has been revealed its vitamins (riboflavin, niacin, acid ascorbic and carotenoids and tocopherol), polyphenols (epigallocatechin, procyanidin, catechin and rutin), polysaccharides (hemicellulose, cellulose, gum and pectin) and flavonoids (such as *quercetin* isomers) components have potential chemoprotective effects such as antihyperlipidemic, antidiabetic, neuroprotective, radical scavenger and anti-fatigue action. In fact, mixture content of both lipophilic and hydrophilic antioxidant components in this plant enables it to scavenge radicals in lipid and aqueous phase. Its flavonoids and vitamin C, synergistically, protect low density lipoprotein oxidation. Furthermore, numerous researches have confirmed that okra^’^s peel and seed powder could elevate total antioxidant status of kidney, pancreas and liver of diabetic rats. Okra’s polysaccharides constituents are responsible to maintain blood glucose level in normal range through controlling sugar absorption from the small intestine (3,4).

Several studies have conducted to examine antidiabetic effects of okra on diabetic animal models. In the study of Sabitha et al, on streptozotocin induced diabetic rats, was investigate antihyperlipidemic and antihyperglycemia properties of peel and seed extract of *Abelmoschus esculentus*. They reported, diabetic rates who received 100 and 200 mg/kg okra^’^s peel and seed extract, compared of diabetic control rats indicated significant alleviation in glucose and HbA1c levels, returned triglyceride and cholesterol level to normal range (2).

More recent investigations, showed the beneficial impacts of okra seed and mucilage on improving histopathological changes and biochemical parameters in preclinical studies. These studies found, the effectivity of okra seed extract on reduction of total cholesterol, lipoproteins containing cholesterol, triglyceride and glucose levels and enhancement of serum insulin level. Interestingly, seed and mucilage of okra treated diabetic rats may also attenuate inflammation and dysfunction of β-cells of Langerhans islets in histopathological examination, in addition to biochemical parameters improvements. Thus it seems that, okra seed extract had a protective role against inflamed pancreatic β-cells through its both antioxidant and anti-inflammatory activities (5).

Similarly, a study conducted by Rafieian et al reported beneficial effects of consumption of *Hibiscus esculentus* powder in blood glucose, triglyceride and total cholesterol levels in Alloxan induced diabetic rats that might are associated with antioxidant components (6).

Hence, these presented data indicates nutritional importance of *Hibiscus esculentus* in health protection and improvement of glycemia and hyperlipidemia induced diabetic complications. *Hibiscus esculentus* consumption can be as a routine therapeutic application for diabetic individuals.

## Authors’ contribution

FDS is the single author of the paper.

## Conflicts of interest

The author declared no competing interests.

## Ethical considerations

Ethical issues (including plagiarism, data fabrication, double publication) have been completely observed by the author.

## Funding/Support

None.
